# A model for bringing TB expertise to HIV providers: Medical consultations to the CDC-funded Regional Tuberculosis Training and Medical Consultation Centers, 2013–2017

**DOI:** 10.1371/journal.pone.0236933

**Published:** 2020-08-31

**Authors:** Robyn Fernando, Ashley C. McDowell, Rajita Bhavaraju, Henry Fraimow, John W. Wilson, Lisa Armitige, Connie Haley, Neela D. Goswami

**Affiliations:** 1 Department of Epidemiology, Rollins School of Public Health, Emory University, Atlanta, Georgia, United States of America; 2 Emory University School of Medicine, Atlanta, Georgia, United States of America; 3 Global TB Institute at Rutgers, The State University of New Jersey, Newark, New Jersey, United States of America; 4 Mayo Clinic Center for Tuberculosis, Rochester, Minnesota, United States of America; 5 Heartland National TB Center, San Antonio, Texas, United States of America; 6 University of Florida, Gainesville, Florida, United States of America; 7 Division of Tuberculosis Elimination, NCHHSTP, Centers for Disease Control and Prevention, Atlanta, Georgia, United States of America; University of the Witwatersrand, SOUTH AFRICA

## Abstract

**Background:**

Persons living with human immunodeficiency virus (HIV) are at a greater risk of developing tuberculosis (TB) compared to people without HIV and of developing complications due to the complexity of TB/HIV coinfection management.

**Methods:**

During 2013–2017, the Centers for Disease Control and Prevention (CDC) funded 5 TB Regional Training and Medical Consultation Centers (RTMCCs) (now known as TB Centers of Excellence or COEs) to provide medical consultation to providers for TB disease and latent TB infection (LTBI), with data entered into a Medical Consultation Database (MCD). Descriptive analyses of TB/HIV-related consultations were conducted using SAS® software, version [9.4] to determine the distribution of year of consultation, medical setting and provider type, frequency of consultations regarding a pediatric (<18 years) patient, and to categorize key concepts and themes arising within consultation queries and medical consultant responses.

**Results:**

Of 14,586 consultations captured by the MCD in 2013–2017, 544 (4%) were categorized as TB/HIV-related, with 100 (18%) received in 2013, 129 (24%) in 2014, 104 (19%) in 2015, 117 (22%) in 2016, and 94 (17%) in 2017. Most TB/HIV consultations came from nurses (54%) or physicians (43%) and from local (65%) or state health departments (10%). Only 17 (3%) of HIV-related consultations involved pediatric cases. Off the 544 TB/HIV consultations, 347 (64%) concerned the appropriate treatment regimen for TB/HIV or LTBI/HIV for a patient on or not on antiretroviral therapy (ART).

**Conclusions:**

The data support a clear and ongoing gap in areas of specialized HIV knowledge by TB experts that could be supplemented with proactive educational outreach. The specific categories of TB/HIV inquiries captured by this analysis are strategically informing future targeted training and educational activities planned by the CDC TB Centers of Excellence, as well as guiding HIV educational efforts at regional and national TB meetings.

## Introduction

Tuberculosis (TB) is the most common opportunistic infection among people living with human immunodeficiency virus (HIV) globally [1]. In the United States (US), there were 8,934 reported cases of TB in 2017. Of the 7,945 (89%) TB patients who had HIV-testing performed, 439 (5.5%) had HIV coinfection [2]. Although recent rates of new TB/HIV diagnoses have declined, individuals with HIV are still at greater risk of developing TB disease compared to those without HIV; additionally, these patients are at increased risk of developing complications due to the complexity of managing TB/HIV coinfection [3]. Inappropriate treatment of HIV and/or TB disease may lead to increased disease morbidity and mortality [4, 5]. The Department of Health and Human Services (DHHS) frequently updates opportunistic infections guidelines, which aid providers in managing the complexities of TB/HIV and LTBI/HIV coinfection [3]. Currently, these guidelines are undergoing revision.

Common challenges encountered in the treatment of TB/HIV coinfection include drug interactions between antiretroviral therapy (ART) agents and anti-TB medications, particularly rifampin and other rifamycins, a class of drugs that are an essential component of anti-TB regimens. Potential problems resulting from co-administration of anti-TB therapy and HIV can include: treatment failure or relapse, drug-induced liver injury, cutaneous adverse drug reactions, TB-associated immune reconstitution inflammatory syndrome (TB-IRIS), and death [6, 7].

Many providers for TB also express concerns surrounding when to start TB therapy and/or ART for persons with newly diagnosed or previously untreated HIV infection [3, 8]. Data are mixed with regard to ART initiation after receiving TB treatment among those with lower CD4 cell counts (<200 cell/mm^3^), but DHHS advises that ART should not be withheld until TB treatment completion [3, 8]. The Strategic Timing of Antiretroviral Treatment (START) study found that early treatment with ART improves health outcomes for people with HIV, including those coinfected with TB; however, each case should be thoroughly examined on an individual basis [9–11]. There are also challenges with optimal management of latent TB infection (LTBI) in HIV patients, who are a group at high priority for treatment of LTBI because of increased risk for progression to TB disease if LTBI is untreated [3].

Due to the intricacy of treating TB/HIV and the risk of complications of both infections, medical professionals treating a patient with TB/HIV coinfection should seek medical consultation from experts trained in the subject matter to ensure proper management of both diseases [3, 8, 12, 13]. This is especially true for challenging cases such as and TB/HIV coinfection during pregnancy or HIV and multidrug-resistant TB (MDR-TB) or extensively drug-resistant TB (XDR-TB) coinfection.

In 2013–2017, the Centers for Disease Control and Prevention (CDC) funded 5 TB Regional Training and Medical Consultation Centers (RTMCCs) now known as TB Centers of Excellence or COEs. These Centers provide medical consultation to healthcare providers caring for patients with TB disease and LTBI with consultation data entered into a Medical Consultation Database (MCD) [14]. The CDC-funded Centers during this time-period included Curry International Tuberculosis Center (CITC), New Jersey Medical School Global Tuberculosis Institute (GTBI), Heartland National Tuberculosis Center (HNTC), Southeastern National Tuberculosis Center (SNTC), and Mayo Clinic Center for Tuberculosis (MCCT). In addition to medical consultation services, the COEs support domestic TB control and prevention efforts through education and training initiatives. The two main medical consultation goals of the TB COEs are: 1) to provide rapid expert medical consultation for TB to health departments, hospitals, medical clinics, and private medical providers throughout the United States; and 2) to build local capacity for medical consultation, continuous educational training, and development of best practices in TB patient care nationally, including various challenging TB subspecialty topics, such as TB/HIV coinfection.

The aim of this evaluation was to analyze medical consultations provided by the COEs between 2013 and 2017 to highlight common consultation questions and concerns from providers regarding care of TB/HIV coinfected patients. We also characterized consultations to evaluate how and by whom the consultation services were being employed for patients coinfected with TB/HIV.

## Methods

### Study design and population

From January 2013 through December 2017, each one of the 5 COEs was assigned to supply staff for medical consultations so that coverage was provided on a regional basis for all 50 US states and 5 US territories (American Samoa, Guam, the Northern Mariana Islands, Puerto Rico, and the U.S. Virgin Islands) [15]. A dataset of medical consultations was compiled and analyzed, as previously described [14], and a subset of consultations for TB/HIV were used for this, both qualitative and quantitative, retrospective cohort analysis [15].

This project was determined by CDC and Emory University to be program evaluation rather than human subjects research and, therefore, did not require Institutional Review Board evaluation.

### Data collection

Health departments, hospitals, medical clinics, and private medical providers throughout the US submitted requests for medical consultation either electronically or by telephone to one of the COEs in 2013–2017.

Standardized variables collected for all consultations included: occupation and clinical setting of calling provider, state of provider practice, pediatric vs adult patient, nature of consultation by sub-topic selected by the caller or medical consultant (MC). Sub-topic options for selection included: adverse effects, case management, contact investigation, diagnosis/lab testing, drug resistance, TB/HIV, legal issues, LTBI, MDR/XDR, nontuberculous mycobacteria (NTM), pharmacology, program/policy, TB disease, transmission/infection control, tuberculin skin tests/interferon-gamma release assays (TST/IGRA) or other. More than one sub-topic could be checked. The original inquiry and MC-provided response were also collected and reviewed. All patient information was de-identified prior to entry to the MCD.

### Data analysis

Of all consultations provided by the 5 COEs during 2013–2017, only those that had the subtopic box “TB/HIV” marked were reviewed. Consultations were excluded from analysis if they were not comprehensible due to a text transcription error in the MCD, did not reference an HIV-positive patient, or had an unclear question or response.

Quantitative analyses using SAS® software, version [9.4] were used to determine the number of consultations originating from each type of medical setting and medical provider and whether the consultation involved a pediatric (<18 years) patient [16].

Descriptive analyses were used to produce a summary of key concepts and themes arising within consultation queries and medical consultant responses. A codebook was developed deductively and inductively from analyzing common themes and sub-themes from a random sample of 100 consultations using the random number generating function in Excel. The codebook was refined through group discussions among team members. Remaining consultations were then coded individually by one member of the study team (RF) through an iterative process of reading consultations using the final version of the codebook and reviewing with other members of the study team.

Quantitative analyses using SAS® software, including frequency and summary procedures, were used to determine the number of consultations that conformed to coded question and answer theme(s) and sub-theme(s). For the analysis of sub-themes, a consultation that addressed multiple sub-themes was counted multiple times. In all other analyses, a consultation was counted only once.

## Results

### Quantitative

**Fig 1:**
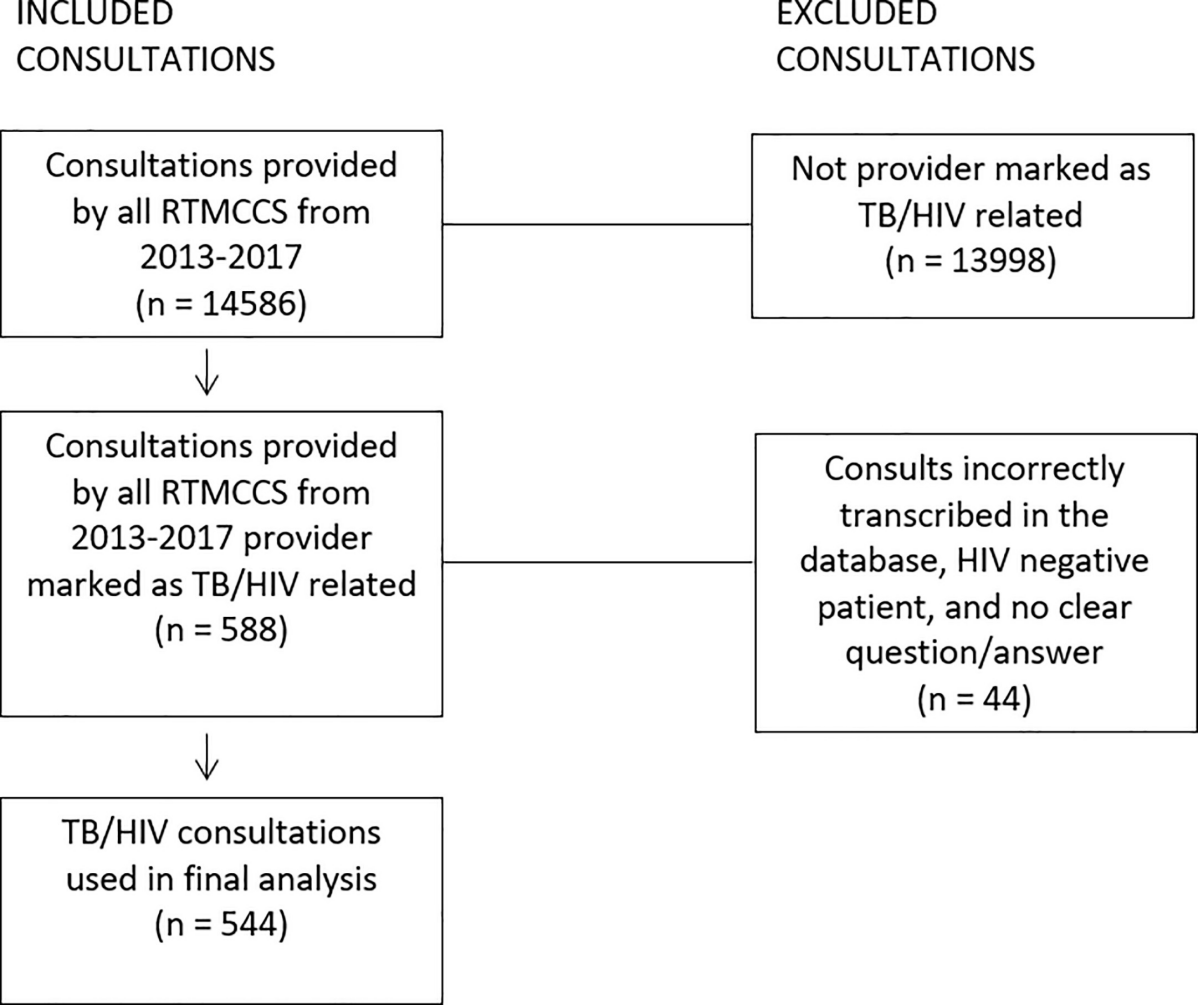
Inclusion and exclusion criteria for analysis.

Over the 5-year period, TB COEs provided 14,586 consultations that were captured in the MCD. Of these, 588 were marked as TB/HIV-related from the checked subtopic box. Of the 588 TB/HIV consultations, 44 were excluded due to not meeting the inclusion criteria: 36 were not properly transcribed by the MCD, 6 requested consultation for HIV-negative patients, and 2 had no clear question/response (Fig 1).

In final analysis, 544 TB/HIV consultations were used, representing 4% of all consultation requests received by the 5 COEs during the funding period, as shown in Table 1. The annual number of TB/HIV consultations received remained consistent throughout the period: 100 consultations were received in 2013, 129 in 2014, 104 in 2015, 117 in 2016, and 94 in 2017. The majority of TB/HIV consultations came from nurses (54%) and physicians (43%). Providers requesting consultations most commonly worked at local (65%) or state health departments (10%), followed by hospitals (8%), and academic institutions (4%). Only 3% of HIV-related consultations involved pediatric cases.

**Table 1:** Summary of TB/HIV consultations^a^ provided by all Regional Training and Medical Consultation Centers (RTMCCs)^b^, 2013–2017.

Characteristic	Consultations, no.	% of Total Consults
**Total consultations**	544	100.0
**Year of consultation**		
2013	100	18.4
2014	129	23.7
2015	104	19.1
2016	117	21.5
2017	94	17.3
**Occupation of consulter**		
Nurse	293	53.9
Physician	235	43.2
Other	16	2.9
**Setting**		
Academic institution	23	4.0
Community health center	8	1.0
Corrections facility	9	2.0
HIV facility	4	1.0
Hospital	45	8.0
International Organization for Migration (IOM)	3	0.6
Local health department	356	65.4
Private practice	10	1.8
Regional health office	19	3.5
State health department	57	10.5
Substance abuse center	2	0.4
Other	8	1.8
**Pediatric**^**c**^		
No	527	96.9
Yes	17	3.1

^a.^ 544 consultations received by the RTMCCS from 2013–2017 met the inclusion criteria.

^b.^ The 5 RTMCCs (now currently known as TB Centers of Excellence or COEs) which provided consults from 2013–2017 were: Curry International Tuberculosis Center, New Jersey Medical School Global Tuberculosis Institute, Heartland National Tuberculosis Center, and Southeastern National Tuberculosis Center, Mayo Clinic Center for Tuberculosis (Mayo Clinic is not a current COE).

^c.^ Cases <18 years of age.

### Qualitative

**Fig 2:**
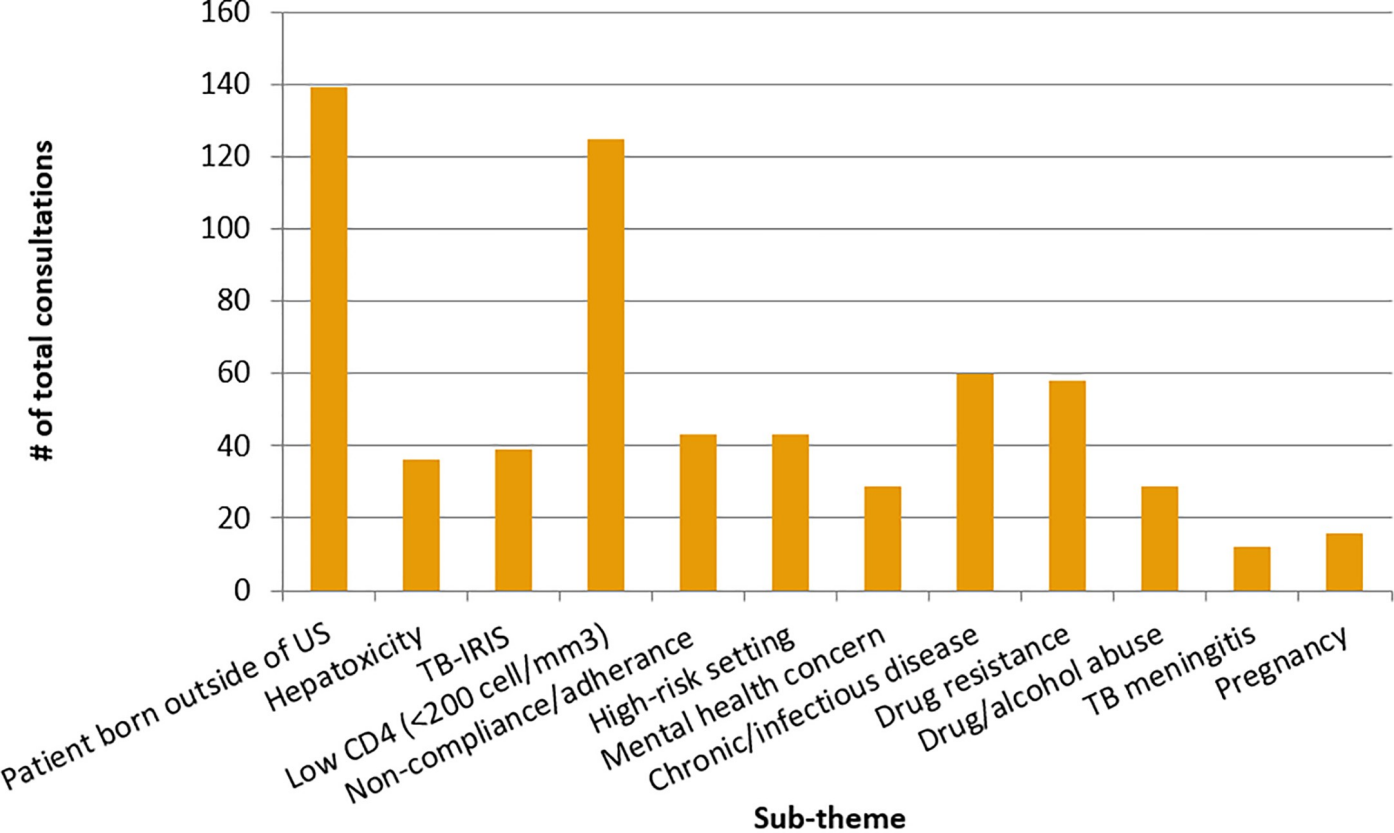
Sub-themes among consultation inquiries received by all Regional Training and Medical Consultation Centers (RTMCCs) between 1/1/13 and 12/31/17, not mutually exclusive. a. TB-associated immune reconstitution inflammatory syndrome. b. <200 cell/mm^3^.

The principal four thematic areas for which TB/HIV consultation was provided were (1) determining the appropriate TB/HIV treatment regimen (51%); (2) considering TB/HIV regimen adjustment or cessation to avoid toxicity, drug interactions, or treatment complications (16%); (3) determining the appropriate LTBI/HIV treatment regimen for a patient (12%); and (4) evaluating the need for further testing or laboratory results before starting or amending a current treatment (7%), shown in Table 2. Consultations were further categorized into 13 different sub-theme topic areas, which were not mutually exclusive (Fig 2). Over a quarter of consultations (26%) involved a patient who was born outside of the United States, 23% involved patients with low CD4 counts (<200 cells/mm^3^), 11% involved questions about concurrent chronic or infectious disease, and 11% mentioned some type of anti-TB drug-resistance, either suspected or confirmed, as shown in Table 3. Further discussion of these main thematic areas is given in each of the sub-sections below.

**Table 2:** Example of codebook.

Full description	Theme shortcut (code)	Key words
**Main Question Theme**		
Appropriate TB/HIV treatment regimen	1	regimen, treatment, tuberculosis, active
Appropriate LTBI/HIV treatment regimen	2	LTBI, latent, treatment
Timing of ART implementation for a newly diagnosed HIV infection for someone who is being treated for TB/LTBI	3	ARV, anti-retroviral, timing
TB/HIV regimen adjustment or cessation to avoid toxicity, drug interactions, or treatment complications	4	complications, symptoms, toxicity
Appropriate treatment regimen for TB/HIV + another chronic disease	5	diabetes, disease, hepatitis
Isolation concerns	6	isolation, released, respiratory
Need for further testing or laboratory results before starting or amending a current treatment	7	repeat, wait, before, additional
Suspected anti-TB drug-resistance	8	resistance
Other	9	Other
**Answer Theme**		
The appropriate treatment regimen was provided based on the information given or multiple hypothetical situations	1	treatment, regimen
The medical consultant referenced or provided a link to the CDC or DHHS TB/HIV guidelines	2	DHHS, guidelines, aidsinfo
The medical consultant requested more information before making a recommendation	3	questions, follow-up, incomplete
Other	4	other

**Table 3:** Summary of main question themes among TB/HIV consultations^a^ received by all Regional Training And Medical Consultation Centers (RTMCCs)^b^, 2013–2017.

	Consultations, no.	% of Total Consults
**Total consultations**	544	
**Main question theme**^**c**^		
Appropriate TB/HIV treatment regimen	280	51.5
TB/HIV regimen adjustment or cessation to avoid toxicity, drug interactions, or treatment complications	88	16.2
Appropriate LTBI/HIV treatment regimen	67	12.3
Need for further testing or laboratory results before starting or amending a current treatment	38	7.0
Appropriate treatment regimen for TB/HIV and another chronic disease	22	4.0
Suspected anti-tuberculosis drug resistance	15	2.8
Other	14	2.6
Isolation concerns	11	2.0
Timing of ART implementation for a newly diagnosed HIV infection for someone who is being treated for TB/LTBI	9	1.7

^a.^ 544 consultations received by the RTMCCS from 2013–2017 met the inclusion criteria to be used in final analysis. Refer to Fig 1 for inclusion/exclusion criteria.

^b.^ The 5 RTMCCs (now currently known as TB Centers of Excellence or COEs) which provided consults from 2013–2017 were: Curry International Tuberculosis Center, New Jersey Medical School Global Tuberculosis Institute, Heartland National Tuberculosis Center, and Southeastern National Tuberculosis Center, Mayo Clinic Center for Tuberculosis (Mayo Clinic is not a current COE).

^c.^ Main question themes were mutually exclusive.

Beyond the four principal thematic areas, five less frequently coded themes were identified contributing, in sum, to 14% total consultations. These themes included appropriate treatment regimen for TB/HIV and another chronic disease (4%); suspected anti-tuberculosis drug-resistance (3%); isolation concerns (2%); timing of ART implementation for a newly diagnosed HIV infection from a patient being treated for TB or LTBI (2%); and other, which encompassed those consultations not fitting another category (3%).

#### Appropriate TB/HIV treatment regimens

The majority of TB/HIV consultations (n = 280, 51%) received by the COEs from 2013–2017 regarded the appropriate treatment regimen for a TB/HIV patient with or without concurrent ART use. The most commonly coded subtheme within this category was low CD4 count (n = 95). Other commonly coded subthemes within this thematic area included drug resistance (n = 28); other chronic or infectious diseases (n = 22); and high-risk setting, including homelessness and incarceration (n = 22).

One consultation within this category related to a coinfected patient with additional hepatitis C infection who was a contact to a drug-resistant patient while incarcerated. The patient was awaiting susceptibility results, and the provider was requesting assistance regarding initial anti-tuberculosis treatment regimen.

#### TB/HIV regimen adjustment or cessation to avoid toxicity, drug interactions, or treatment complications

Most questions pertaining to dose adjustments or treatment cessation in order to avoid toxicity, drug interactions, or treatment complications (n = 88, 16%) related to rifamycin (rifampin or rifabutin) use in combination with a variety of ART medications. Within the 88 consultations coded to this theme, many questions expressed concern about the management of hepatotoxicity (n = 15), TB-immune reconstitution inflammatory syndrome (TB-IRIS) (n = 18), and other chronic or infectious diseases (n = 12), which were commonly coded as subthemes. The most commonly coded subtheme within this category was low CD4 count (n = 29).

MC responses, detailed in Table 4, often referenced official DHHS guidelines (n = 61, 11%)—frequently specifically citing drug interaction tables (Tables 21a-e, available at https://aidsinfo.nih.gov/contentfiles/lvguidelines/adultandadolescentgl.pdf), which outline treatment options including acceptable drug combinations [3, 17]. 76 (14%) responses described the need to dose anti-tuberculous agents at least three times per week (TIW dosing). Finally, 40 (7%) MC responses recommended substituting rifabutin for rifampin in a patient’s current regimen in order to avoid drug interactions with concurrently prescribed ART medications.

**Table 4:** Summary of main answer themes among TB/HIV consultations^a^ provided by all Regional Training And Medical Consultation Centers (RTMCCs)^b^, 2013–2017.

	Consultations, no.	% of Total Consults
**Total consultations**	544	
**Answer theme**^**c**^		
The appropriate treatment regimen was provided based on the information given and:	391	71.9
The medical consultant (MC) referenced or provided a link to the official DHHS^d^ TB/HIV guidelines or:	28	5.1
The MC requested more laboratories or information	76	14.0
All 3 of the above	2	0.4
The MC referenced or provided a link to the official DHHS TB/HIV guidelines	10	1.8
The respondent requested more labs/information	25	4.6
Other	12	2.2

^a.^ 544 consultations received by the RTMCCS from 2013–2017 met the inclusion criteria to be used in final analysis. Refer to Fig 1 for inclusion/exclusion criteria.

^b.^ The 5 RTMCCs (now currently known as TB Centers of Excellence or COEs) which provided consults from 2013–2017 were: Curry International Tuberculosis Center, New Jersey Medical School Global Tuberculosis Institute, Heartland National Tuberculosis Center, and Southeastern National Tuberculosis Center, Mayo Clinic Center for Tuberculosis (Mayo Clinic is not a current COE).

^c.^ Answer themes were mutually exclusive.

^d.^ Department of Health and Human Services.

#### Appropriate LTBI/HIV treatment regimens

Consultations that asked for the appropriate treatment regimen for LTBI/HIV treatment (n = 67, 12%) tended to cover a wide variety of clinical subjects. However, common threads within this category are evidenced in the most commonly coded subthemes including other chronic or infectious disease (n = 10), high-risk setting (n = 9), drug resistance (n = 7), and non-compliance (n = 6).

Those consultations regarding LTBI/HIV treatment regimens often involved questions about anti-TB medication timing, implementation, and duration of treatment. For example, one consultation request related to the timing of initiation and the necessary duration of LTBI treatment in a patient with transaminitis secondary to his or her ART.

#### Need for further testing or laboratory results before starting or amending a current treatment

Consultation queries received by the COEs that asked about the need for obtaining further testing or laboratory results before the patient started treatment (n = 38, 7%) were less analogous and more individually case-specific than questions that fell under other main themes. The most frequently coded subtheme within this category was low CD4 count (n = 8), followed by high-risk setting (n = 6), non-compliance (n = 4), and other chronic or infectious disease (n = 4).

One example within this theme involved a consultation request made regarding an asymptomatic patient without TB risk factors who was recently found to have a positive IGRA result followed by a negative chest x-ray. The question involved further management of this patient including the need for a repeat IGRA level.

Another consultation was made regarding a symptomatic patient living in a homeless shelter. The recommendation was to expedite diagnostic labs, preferentially through a hospital admission, in order to more quickly introduce a treatment regimen and reduce infectivity within his or her high-risk setting.

## Discussion

Our findings demonstrate that consultation services provided by the COEs were consistently utilized by healthcare providers regarding TB/HIV from 2013–2017. Local health departments were the main users of the consultation services, with the majority of questions concerning the appropriate treatment regimen for a TB/HIV coinfected patient with or without concurrent ART (51%). We observed more consultations from public health providers than from non-public health entities, such as substance abuse centers, private practices, HIV facilities, and correctional facilities. This is not surprising given the similar health department predominance shown in a recent evaluation of all consultations made to the COEs between 2013 and 2018 [14]. As TB/HIV coinfections can be seen and treated at all of these types of facilities, it could be beneficial to market CDC-funded consultation services to non-public health practices in order to raise awareness of the service.

### HIV and TB disease

The major themes specified in this study indicate common foci of TB/HIV-related consultation. The most common theme of consultation was that of appropriate TB/HIV treatment regimen. Notably, many inquiring providers asked about treatment recommendations for coinfected patients with more complex or difficult to manage cases: those focusing on coinfection in the setting of AIDS (CD4 <200), potential medication resistance, one or more patient comorbidity, and high-risk patient setting including homelessness and incarceration. Common subthemes within the category of TB/HIV regimen adjustment or cessation to avoid toxicity, drug interactions, or treatment complications suggest that commonly encountered treatment complications include TB-IRIS and hepatotoxicity and that providers may frequently have concerns regarding continued management in patients with pre-existing comorbidities or advanced HIV disease.

While there were still consultation questions regarding less complicated patients, these findings may suggest a need for education regarding management of patients with complicated coinfection, particularly those with medication toxicities, advanced HIV disease, and pre-existing medical conditions, and for raising awareness on the existence of guidelines for the management of TB/HIV coinfection [3, 8]. This is especially important given the rapidly expanding arsenal of ART medications, including new combination pills recommended as first-line therapy, as many of these have limitations for co-administration with some anti-TB therapies [3].

### HIV and LTBI

Consultations relating to appropriate treatment of LTBI/HIV often referenced medication resistance, other chronic or infectious diseases, medication non-compliance, and high-risk patient setting. Thus, while providers managing TB/HIV coinfection may often request consultation due to complicated cases, providers seeking advice for patients with LTBI/HIV coinfection are more often concerned with the potential for incomplete or ineffective treatment and the risk of contact infection.

### Necessary further evaluation for LTBI or TB disease

Finally, consultations within the category need for further testing or laboratory results before starting or amending a current treatment most commonly included the subthemes of high-risk patient setting, medication non-compliance, other chronic or infectious diseases, and low CD4 count. These results may indicate that consultants are commonly using clinical and contextual information about patients in order to tailor coinfection treatment and highlight the utility of expert consultants in management of TB/HIV coinfection.

This study had several limitations. Only consultations that were marked as TB/HIV and properly transcribed were reviewed for analysis. Key word searches to locate other TB/HIV-related queries that may have been misclassified due to user error or not specifically noted if there were multiple concerns being addressed in a single consultation were considered but not performed due to resource restrictions, so the percentage of the total consultations that are TB/HIV-related from all consultations from 2013–2017 may be higher. There was heterogeneity in how each COE entered consultations into the MCD, which may have resulted in incomplete capture of consultations provided during the study period. Since data were de-identified prior to being entered in the MCD, we were not able to identify how many of the consultations in the MCD were follow-up consultations concerning the same patient, which may have affected the distribution of question and answer themes. We also did not have access to outcome data on the patients to determine if the consultation services meaningfully impacted patient outcomes or provider practices. Additionally, we were unable to separate nurse practitioners from nurses in the dataset.

Regardless of these limitations, a relatively stable number of TB/HIV-related consultations were received each year, signifying that consultation services were utilized consistently throughout the study period. With TB/HIV-coinfection on the decline, expertise among community providers may wane, making private provider relationships with health departments and medical consultation services even more important. The data support a clear and ongoing gap in areas of specialized HIV knowledge by TB experts that could be supplemented with proactive educational outreach. The specific categories of TB/HIV inquiries captured by this analysis are strategically informing future targeted training and educational activities planned by the CDC TB Centers of Excellence, as well as being used to guide HIV educational efforts at regional and national TB meetings. Strengthening the knowledge base of the TB provider and his or her confidence in this expertise will inevitably improve his or her evidence-based care of the patient with TB or LTBI and HIV coinfection.

As treating TB/HIV patients can often be complicated and official DHHS guidelines on TB/HIV coinfection are continuously updated, accessing the medical consultation services provided by public health consultants and COEs may help ensure TB/HIV coinfected patients receive up-to-date care. It is vital that both HIV and TB providers be a part of the multidisciplinary care team for an individual with TB/HIV coinfection. Experts in TB care are not necessarily experts in HIV care, and vice versa. Medical consultation services provide a valuable bridge in this area.
